# Methylated promoter DNA of *CDO1* gene and preoperative serum CA19-9 are prognostic biomarkers in primary extrahepatic cholangiocarcinoma

**DOI:** 10.1371/journal.pone.0205864

**Published:** 2018-10-16

**Authors:** Shuji Nakamoto, Yusuke Kumamoto, Kazuharu Igarashi, Yoshiki Fujiyama, Nobuyuki Nishizawa, Shigenori Ei, Hiroshi Tajima, Takashi Kaizu, Masahiko Watanabe, Keishi Yamashita

**Affiliations:** 1 Department of Surgery, Kitasato University Hospital, Kitasato, Minami-ku, Sagamihara, Kanagawa, Japan; 2 Division of Advanced Surgical Oncology, Research and Development Center for New Medical Frontiers, Kitasato University School of Medicine, Kitasato, Minami-ku, Sagamihara, Kanagawa, Japan; University of South Alabama Mitchell Cancer Institute, UNITED STATES

## Abstract

**Background:**

Promoter DNA methylation of *Cysteine dioxygenase type1 (CDO1)* gene has been clarified as a molecular diagnostic and prognostic indicator in various human cancers. The aim of this study is to investigate the clinical relevance of *CDO1* methylation in primary biliary tract cancer (BTC).

**Methods:**

*CDO1* DNA methylation was assessed by quantitative methylation-specific PCR in 108 BTC tumor tissues and 101 corresponding normal tissues. BTC was composed of extrahepatic cholangiocarcinoma (EHCC) (n = 81) and ampullary carcinoma (AC) (n = 27).

**Results:**

The *CDO1* methylation value in the tumor tissues was significantly higher than that in the corresponding normal tissues (p<0.0001). The overall survival (OS) in EHCC patients with hypermethylation was poorer than those with hypomethylation (p = 0.0018), whereas there was no significant difference in AC patients. Multivariate analysis identified that *CDO1* hypermethylation, preoperative serum CA19-9 and perineural invasion were independent prognostic factors in EHCC. The EHCC patients with *CDO1* hypermethylation exhibited more dismal prognosis than those with hypomethylation even in low group of CA19-9 level (p = 0.0006).

**Conclusions:**

Our study provided evidence that promoter DNA methylation of *CDO1* gene could be an excellent molecular diagnostic and prognostic biomarker in primary EHCC. The combination of *CDO1* methylation and preoperative serum CA19-9 effectively enriched EHCC patients who showed the most dismal prognosis. These markers would be beneficial for clinical clarification of the optimal strategies in EHCC.

## Introduction

Biliary tract cancer (BTC) is uncommon malignancy with an unfavorable prognosis. According to cancer statistics, 2017, BTC patients including gallbladder cancer were estimated as new cases of 11,740 and deaths of 3,830 in the United States.[1] Recent studies have reported that the global incidence has shown increasing tendency worldwide.[2,3] BTC is epithelial cancer that arise from biliary tree and can be classified by anatomic location. This classification differs not only from location but epidemiology, origin, treatment and prognosis. Extrahepatic cholangiocarcinoma (EHCC) is the most common type of BTC.[3] Among them, EHCC and ampullary carcinoma (AC) produce similar clinical presentation, and they are often diagnosed by the onset of obstructive jaundice. However, the prognosis of all subtypes of BTC has not improved due to the difficulty of early diagnosis and the limitation of effective treatment. Surgical resection remains the only potentially curative treatment, while many patients have experienced the recurrence. Such poor outcome has prompted interest in the use of adjuvant chemotherapy and radiation therapy. However, there is a paucity of high-quality evidence to support clinical effects of adjuvant therapy following resection in BTC, and patients should be encouraged to participate in clinical trials evaluating new strategies, and molecular biomarkers for the evaluation of BTC are highly demanded in clinical practice, with establishment of optimal guidelines for adjuvant or neoadjuvant therapy.

Epigenetic gene silencing of tumor suppresser genes through promoter DNA hypermethylation is a common feature in human cancers, whereas cancer-specific methylation is a relatively rare event.[4,5] We have developed pharmacologic reversal of epigenetic silencing and thereby uncovered a myriad of transcriptionally repressed genes in human cancers.[6] Using this technique, we have identified novel tumor suppressor gene candidates including the *cysteine dioxygenase type1 (CDO1)* gene. We and others have previously described that aberrant DNA methylation of the *CDO1* promotor region is diagnostic and/or prognostic biomarker in various cancers, such as breast, esophageal, gastric, colorectal, renal, prostate, and gallbladder cancer. [7–16] Additional study has further shown the clinical utility of tumor diagnosis using *CDO1* promoter DNA methylation in an endoscopic retrograde cholangiography (ERCP) solution of BTC as alternate cytology test.[17] Nevertheless, there have no reports with regard to clinico-pathological relevance of *CDO1* methylation in primary BTC.

In the present study, we for the first time investigated the clinico-pathological and prognostic relevance of promotor DNA methylation of the *CDO1* gene assessed by the quantitative PCR in primary BTC.

## Materials and methods

### Patients and tissue samples

This study investigated 108 patients who underwent surgical resection for primary BTC at the Kitasato university hospital, Japan between October 1988 and November 2012. We extracted DNA from 108 tumor tissues and 101 corresponding normal tissues. These tissue samples were collected from all patients who obtained written informed consent to use their pathological specimens. The present study was approved by the Ethics Committee of Kitasato University. Patient characteristics are shown in Table 1. Patients with neoadjuvant chemotherapy was not included.

**Table 1:** Univariate and multivariate analysis of clinico-pathological factors affecting 5-year overall survival in primary BTC.

(A) Extrahepatic cholangiocarcinoma							
**Clinico-pathological parameter**		**Number**	**Univariate analysis**		**Multivariate analysis**		
			**5yOS (%)**	**p value**	HR*	95%CI*	**p value**
Age	≥65 / <65	46 / 35	41 / 55	0.31			
Gender	Male / Female	59 / 22	42 / 64	0.16			
Preoperative jaundice	absence / presence	40 / 41	47 / 47	0.81			
Biliary drainage	absence / presence	13 / 68	64 / 44	0.19			
Preoperative serum CA19-9	≥37 / <37	52 / 29	35 / 74	0.0084	2.3	1.0–5.9	0.047
Preoperative serum CEA	≥5 / <5	4 / 77	0 / 49	0.12			
Tumor location	Bp / Bd	18 / 63	43 / 48	0.79			
Lymphatic permeation	absence / presence	18 / 63	76 / 40	0.0047	1.6	0.52–7.4	0.42
Vascular permeation	absence / presence	25 / 56	59 / 43	0.22			
Portal venous invasion	absence / presence	77 / 4	49 / 25	0.0003			
Arterial system invasion	absence / presence	78 / 3	48 / 33	0.49			
Perineural invasion	absence / presence	22 / 59	80 / 36	0.0021	3.1	1.2–11	0.018
Macroscopic growth pattern	invasive / others	71 / 10	45 / 68	0.25			
Histology	tub1, pap / others	49 / 32	53 / 39	0.23			
Resection status	R0 / R1, 2	43 / 38	57 / 36	0.04	1.4	0.71–2.7	0.34
CDO1 TaqMeth value	≥28.9 / <28.9	22 / 59	22 / 56	0.0018	2.4	1.2–4.7	0.016
the sixth TNM classification							
pT	Tis,T1 / T2 / T3 / T4	10 / 12 / 31 / 28	100 / 56 / 40 / 34	0.01			
pN	absence / presence	43 / 38	66 / 27	0.0002			
pStage				0.016			0.079
	0, IA, IB	15	76		Reference		
	IIA	17	61		1.0	0.25–5.1	
	IIB	21	33		2.0	0.58–9.7	
	III	28	34		3.0	0.90–14	
Operative procedure	PD / Liver resection / others	64 / 14 / 3	49 / 42 / 33	0.64			
Postoperative jaundice	absence / presence	70 /11	47 / 55	0.75			
Postoperative serum CA19-9	≥37 / <37	19 / 62	31 / 52	0.077			
Postoperative serum CEA	≥5 / <5	2 / 79	100 / 46	0.21			
Postoperative chemotherapy	absence / presence	20 / 61	62 / 43	0.34			
(B) Ampullary carcinoma							
**Clinico-pathological parameter**		**Number**	**Univariate analysis**		**Multivariate analysis**		
			**5yOS (%)**	**p value**	HR*	95%CI*	**p value**
Age	≥65 / <65	16 / 11	65 / 82	0.57			
Gender	Male / Female	16 / 11	70 / 81	0.66			
Preoperative jaundice	absence / presence	16 / 11	88 / 61	0.24			
Biliary drainage	absence / presence	4 / 23	100 / 70	0.31			
Preoperative serum CA19-9	≥37 / <37	11 / 16	57 / 88	0.19			
Preoperative serum CEA	≥5 / <5	1 / 26	0 / 76	0.0068		1.5	0.06–20	0.76
Lymphatic permeation	absence / presence	11 / 16	100 / 59	0.036	1.6E+08	0.04-	0.52
Vascular permeation	absence / presence	10 / 17	100 / 60	0.047		1.2E+08	0.03-	0.58
Portal venous invasion	absence / presence	27 / 0	74 / -				
Arterial system invasion	absence / presence	27 / 0	74 / -				
Perineural invasion	absence / presence	21 / 6	86 / 25	0.0008	1.0	0.04–15	0.99
Macroscopic growth pattern	invasive / others	11 / 16	32 / 94	0.014		4.0	0.28–97	0.29
Histology	tub1, pap / others	21 / 6	75 / 67	0.49			
Resection status	R0 / R1, 2	24 / 3	83 / 0	<0.0001	4.3	0.37–101	0.24
CDO1 TaqMeth value	≥28.9 / <28.9	9 / 18	66 / 78	0.89			
the sixth TNM classification							
pT	Tis,T1 / T2 / T3 / T4	9 / 4 / 14 / 0	100 / 100 / 54 / -	0.052			
pN	absence / presence	11 / 16	91 / 65	0.25			
pStage	0, IA, IB / IIA / IIB / III	8 / 3 / 16 / 0	100 / 67 / 65 / -	0.26			
Postoperative jaundice	absence / presence	27 / 0	74 / -				
Postoperative serum CA19-9	≥37 / <37	5 / 22	50 / 82	0.45			
Postoperative serum CEA	≥5 / <5	1 / 26	100 / 73	0.68			
Postoperative chemotherapy	absence / presence	10 / 17	90 / 66	0.29			

*HR: hazard ratio, CI: confidence interval

### DNA extraction and sodium bisulfite conversion

Tissue sections from primary tumors and corresponding normal tissues were stained with hematoxylin and eosin, and dissected under microscope. Genomic DNA was extracted from formalin-fixed paraffin embedded (FFPE) tissues using a QIAamp DNA FFPE Tissue Kit (QIAGEN Sciences, Hilden, Germany). Bisulfite treatment was done according to the manufacturer’s instructions of an EZ DNA Methylation-Gold^TM^ Kit (Zymo Research, Orange, CA).

### Quantitative-methylation-specific PCR (Q-MSP)

Quantitative-TaqMan methylation specific PCR (Q-MSP) was carried out using iQ Supermix (Bio-Rad Laboratories, Hercules, CA) in triplicate on the C1000 Touch^TM^ Thermal Cycler CFX96 Real Time System (Bio-Rad). Bisulfite-treated DNA was amplified by the following PCR conditions: 95°C for 3 min, followed by 40 cycles at 95°C for 20 sec, annealing temperature (60°C) for 30 sec, and 72°C for 30 sec. The sequences of primers and probes are provided in S1 Table.[8–10,12,16] Serial dilutions of bisulfite modified DNA from human colon carcinoma cell line DLD1 was used to construct the calibration curve on each plate as a methylation positive control, and human hepatoblastoma cell line HepG2 was used as a negative control as previous described.[8–10,12,16] The methylation value was defined as a TaqMeth value by the ratio of the amplified signal value of methylated *CDO1* to the value for *β-actin*, which was then multiplied by 100.[7]

### Conventional MSP

Conventional MSP was performed using Platinum Taq DNA Polymerase (Invitrogen) according to the manufacturer’s protocol. Methylated and unmethylated primers for PCR amplification were designed to partially cover the CpG island of *CDO1* promoter region. Primers sequences are also included in S1 Table. The PCR conditions were the same as Q-MSP. The PCR products were separated on 1.5–2.0% agarose gel, then visualized by ethidium bromide staining. Distilled water was used as negative control.

### Immunohistochemistry

Immunostaining was performed on formalin-fixed, paraffin-embedded sections (4 μm thick). Sections were incubated using the anti-CDO1 rabbit polyclonal antibody (dilution of 1:100) (ATLAS ANTIBODIES, Bromma, Sweden). Immune complexes were detected with a Histofine Simple Stain MAX PO (MULTI) (Nichirei, Tokyo, Japan), following the manufacturer’s protocol, and visualized using the 3,3’-diaminobenzidine (DAB) substrate. Sections were counter-stained with Hematoxylin solution.

### Statistical analysis

Continuous variables were analyzed using Mann-Whitney’s U test and Kruskal-Wallis test, as appropriate. Categorical variables were analyzed using *X*^2^ test. Clinico-pathological characteristics and follow up data were evaluated in terms of 5-year overall survival (5yOS). The follow up time was calculated from the date of surgery to death or end-point. 5yOS was estimated by Kaplan-Meier method, and compared using a log-rank test. Variables suggesting potential prognostic factors on univariate analyses were subjected to a multivariate analysis using a Cox proportional-hazards model. P-value <0.05 was considered to indicate statistical significance. All statistical analyses were conducted using the SAS software package (JMP Pro11, SAS Institute, Cary, NC).

## Results

### Promotor DNA methylation level of *CDO1* gene and its correlation with clinico-pathological factors in primary BTC

**Fig 1:**
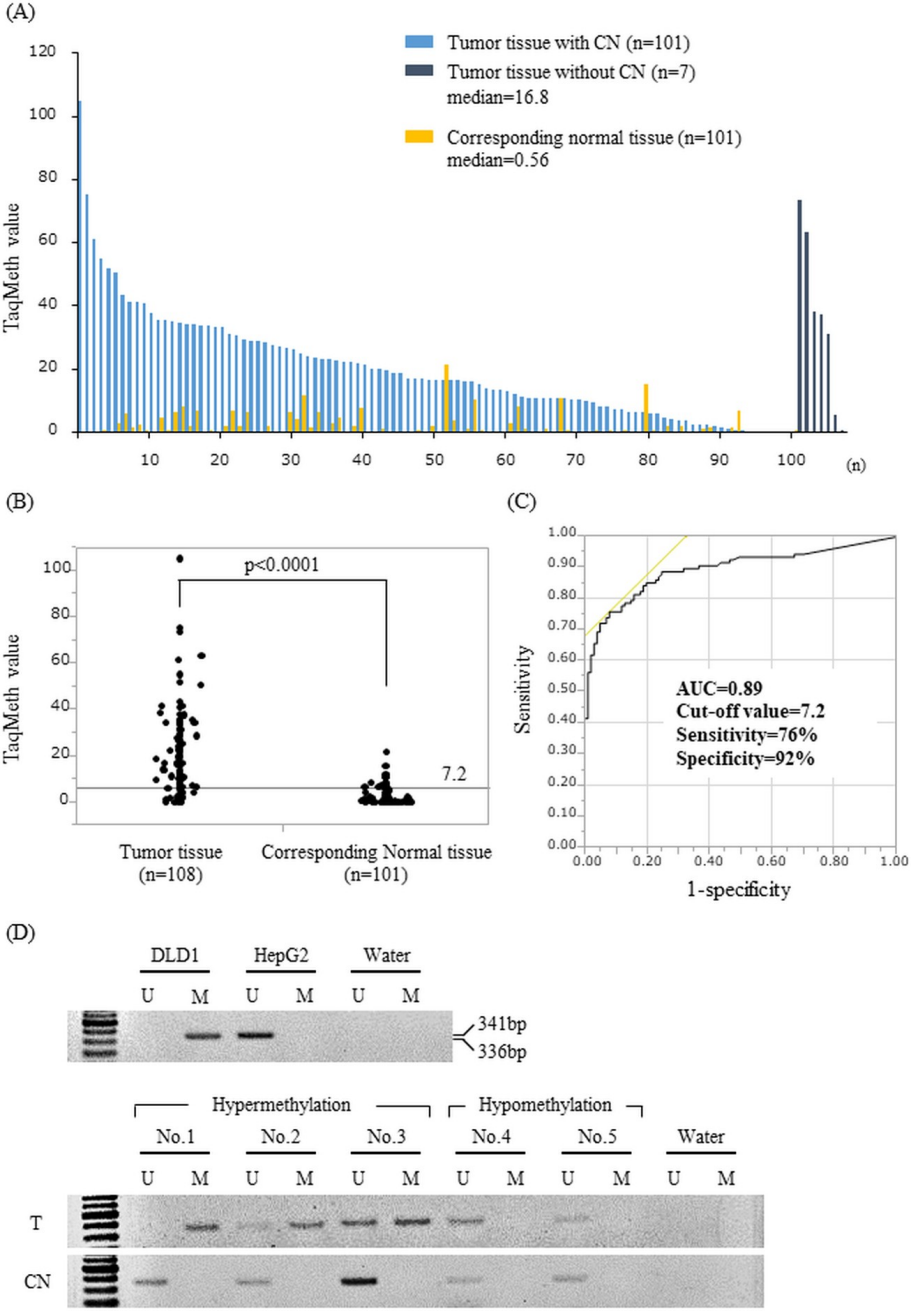
Quantitative assessment of *CDO1* methylation and representative conventional MSP in primary BTC. (A) *CDO1* TaqMeth value of the 108 primary BTC tumor tissues (T) and 101 corresponding normal tissues (CN). The median of T and CN were 16.8 and 0.56, respectively. (B) There was a significant difference in *CDO1* methylation values between T and CN (p< 0.0001). (C) ROC curve of *CDO1* methylation for detection of BTC. Area under the curve (AUC) represents the accuracy in discriminating normal from tumor in term of sensitivity and specificity (p< 0.0001). (D) Representative conventional MSP of *CDO1* gene in T and CN (U: unmethylation, M: methylation).

A total of 108 primary tumor specimens of BTC patients who underwent surgical resection were assessed by Q-MSP to evaluate the clinical relevance of the *CDO1* methylation level. The median TaqMeth value was 16.8, ranging from 0 to 105 in the 108 tumor tissues (T) and 0.56, ranging from 0 to 21.3 in the 101 corresponding normal tissues (CN) (Fig 1A). There was significant difference in *CDO1* methylation value between T and CN (p<0.0001) (Fig 1B). The most optimal cut-off value of 7.2 was calculated from receiver operating characteristic (ROC) analysis for maximizing both sensitivity and specificity of BTC detection as compared to CN, where sensitivity was 76%, and specificity was 92% (Fig 1C). We also confirmed differential methylation with conventional MSP in 10 tissue samples. T and CN are TaqMeth values of 1.2–105 and 0.0–1.4, respectively. T with hypermethylation amplified by methylated primers, but hypomethylation and CN samples were not methylated bands (Fig 1D).

Correlation of each clinico-pathological factor to TaqMeth value of *CDO1* gene in primary BTC was evaluated by Mann-Whitney’s U test or Kruskal-Wallis test. There was significant correlation of *CDO1* TaqMeth value to vascular permeation (p = 0.035). The other clinico-pathological factors showed no significant association with *CDO1* TaqMeth value (S2 Table).

**Fig 2:**
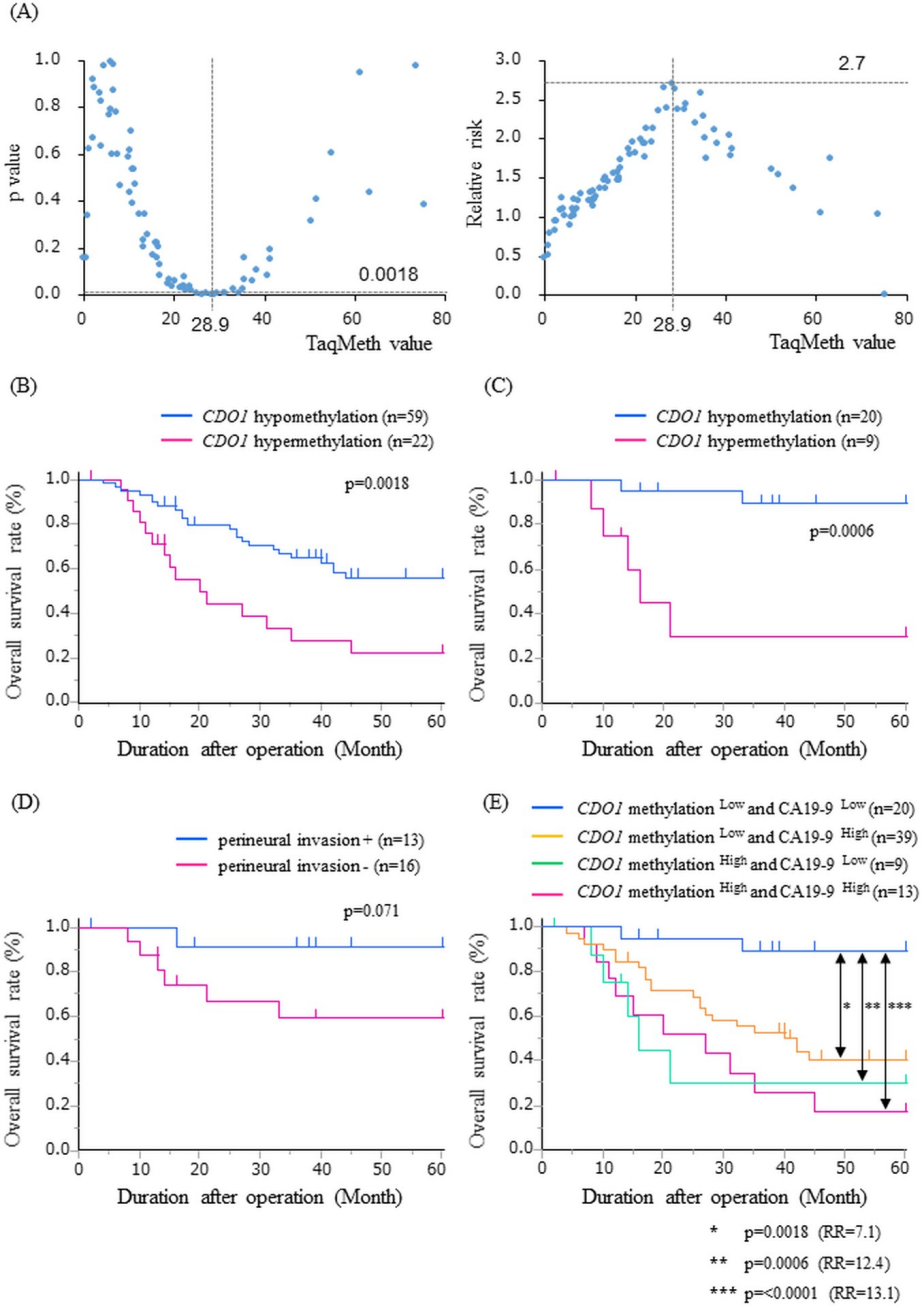
Optimal cut-off value and survival analysis in primary extrahepatic cholangiocarcinoma. (A) Identification of an optimal cut-off value for the prognosis using the log-rank plot analysis. Kaplan-Meier curves for 5-year overall survival stratified by *CDO1* methylation status (B), and *CDO1* methylation status and perineural invasion in low levels of CA19-9 (C), (D). Kaplan-Meier curves for the combination of *CDO1* methylation and CA19-9 (E). RR: relative risk.

We further investigated the correlation of *CDO1* TaqMeth value to OS in primary BTC. To determine the optimal cut-off values for predicting prognosis, we assessed each p value and relative risk by the log rank plot analysis. The most optimal cut-off value was defined as 28.9, which showed the highest relative risk with statistical significance in EHCC (Fig 2A). The EHCC patients with *CDO1* hypermethylation (≥28.9) showed poor prognosis than those with hypomethylation (<28.9) (5yOS: 22% vs 56%, p = 0.0018) (Fig 2B). However, any cut-off values could not represent prognostic stratification in AC (S1 Fig).

### Univariate and multivariate prognostic analysis including *CDO1* promoter DNA methylation status in primary BTC

The clinico-pathological factors related to prognosis were examined separately in 81 EHCC and 27 AC patients, because there was large difference of their prognosis (5yOS: 48% vs 74%, p = 0.029).

Univariate analysis showed that preoperative serum values of CA19-9 (p = 0.0084), lymphatic permeation (p = 0.0047), portal venous invasion (p = 0.0003), perineural invasion (p = 0.0021), resection status (p = 0.04), *CDO1* TaqMeth value (p = 0.0018), pT (p = 0.01), pN (p = 0.0002), and pStage (p = 0.016) were significantly associated with poor prognosis in EHCC. The identified univariate prognostic factors were subjected to multivariate analysis, in which staging factors were excluded, i.e., portal venous invasion, pT and pN, because they were confounding factors for pStage. Multivariate analysis indicated that preoperative serum value of CA19-9 (p = 0.047), perineural invasion (p = 0.018), and *CDO1* TaqMeth value (p = 0.016) were finally remnant independent prognostic factors related to OS in EHCC (Table 1A).

On the other hand, univariate analysis in AC showed preoperative serum value of CEA (p = 0.0068), lymphatic permeation (p = 0.036), vascular permeation (p = 0.047), perineural invasion (p = 0.0008), macroscopic growth pattern (p = 0.014), and resection status (p<0.0001) were significantly poor prognosis. Multivariate analysis revealed that there was no independent factor in AC (Table 1B).

### Correlation of clinico-pathological factors to promoter DNA methylation status of the *CDO1* gene divided by the prognostically optimized cut-off value in primary EHCC

Correlation between clinico-pathological factors and *CDO1* methylation status divided by cut-off value of 28.9 in primary EHCC was determined by a X^2^ test. Preoperative serum CEA was the only significant parameter associated with *CDO1* methylation value (p = 0.027) (Table 2).

**Table 2:** Correlation of clinico-pathological chracterestics and CDO1 methylation in primary EHCC.

Factor category	Clinico-pathological parameter	CDO1 TaqMeth value					
		Low (<28.9)			High (≥28.9)		p value
		No.	%		No.	%	
Preoperative factor	Age	≥65	30	65	16	35	0.08
		<65	29	83	6	17	
	Gender	Male	41	69	18	31	0.27
		Female	18	82	4	18	
	Preoperative jaundice	absence	27	68	13	33	0.29
		presence	32	78	9	22	
	Biliary drainage	absence	9	69	4	31	0.75
		presence	50	74	18	26	
	Preoperative serum CA19-9	≥37	39	75	13	25	0.56
		<37	20	69	9	31	
	Preoperative serum CEA	≥5	1	25	3	75	0.027
		<5	58	75	19	25	
Pathological factor	Tumor location	Bp	11	61	7	39	0.20
		Bd	48	76	15	24	
	Lymphatic permeation	absence	16	89	2	11	0.083
		presence	43	68	20	32	
	Vascular permeation	absence	20	80	5	20	0.33
		presence	39	70	17	30	
	Portal venous invasion	absence	57	74	20	26	0.29
		presence	2	50	2	50	
	Arterial system invasion	absence	56	72	22	28	0.28
		presence	3	100	0	0	
	Perineural invasion	absence	17	77	5	23	0.58
		presence	42	71	17	29	
	Macroscopic growth pattern	invasive	52	73	19	27	0.83
		others	7	70	3	30	
	Histology	tub1,pap	38	78	11	22	0.24
		others	21	66	11	34	
	Resection status	R0	31	72	12	28	0.87
		R1,2	28	74	10	26	
	pT	Tis,T1	9	90	1	10	0.40
		T2	10	83	2	17	
		T3	21	68	10	32	
		T4	19	68	9	32	
	pN	absence	35	81	8	19	0.07
		presence	24	63	14	37	
	pStage	0,IA,IB	14	93	1	7	0.26
		IIA	12	71	5	29	
		IIB	14	67	7	33	
		III	19	68	9	32	
Treatment factor	Operative procedure	PD	49	77	15	23	0.19
(postoperative factor)		Liver resection	9	64	5	36	
		others	1	33	2	67	
	Postoperative jaundice	absence	50	71	20	29	0.47
		presence	9	82	2	18	
	Postoperative serum CA19-9	≥37	11	58	8	42	0.09
		<37	48	77	14	23	
	Postoperative serum CEA	≥5	2	100	0	0	0.38
		<5	57	72	22	28	
	Postoperative chemotherapy	absence	15	75	5	25	0.80
		presence	44	72	17	28	

### Prognostic relevance of *CDO1* TaqMeth value in EHCC

We then examined prognostic relevance of the combination of the independent prognostic factors, *CDO1* methylation and preoperative serum CA19-9 level, and perineural invasion in EHCC. Kaplan-Meier curves showed that patients with *CDO1* hypermethylation showed significantly more dismal prognosis than those with hypomethylation even among the patients with low preoperative serum value of CA19-9 (5yOS: 30% vs 89%, p = 0.0006) (Fig 2C). There was no significant difference between patients with and without perineural invasion in low value of CA19-9 (p = 0.071) (Fig 2D). Finally, EHCC patients with *CDO1* hypomethylation and low preoperative serum value of CA19-9 exhibited significantly much better prognostic outcome rather than those with either *CDO1* hypermethylation or high preoperative value of CA19-9 (Fig 2E).

### Promoter methylation of *CDO1* gene critically affects CDO1 expression in tumor tissues

**Fig 3:**
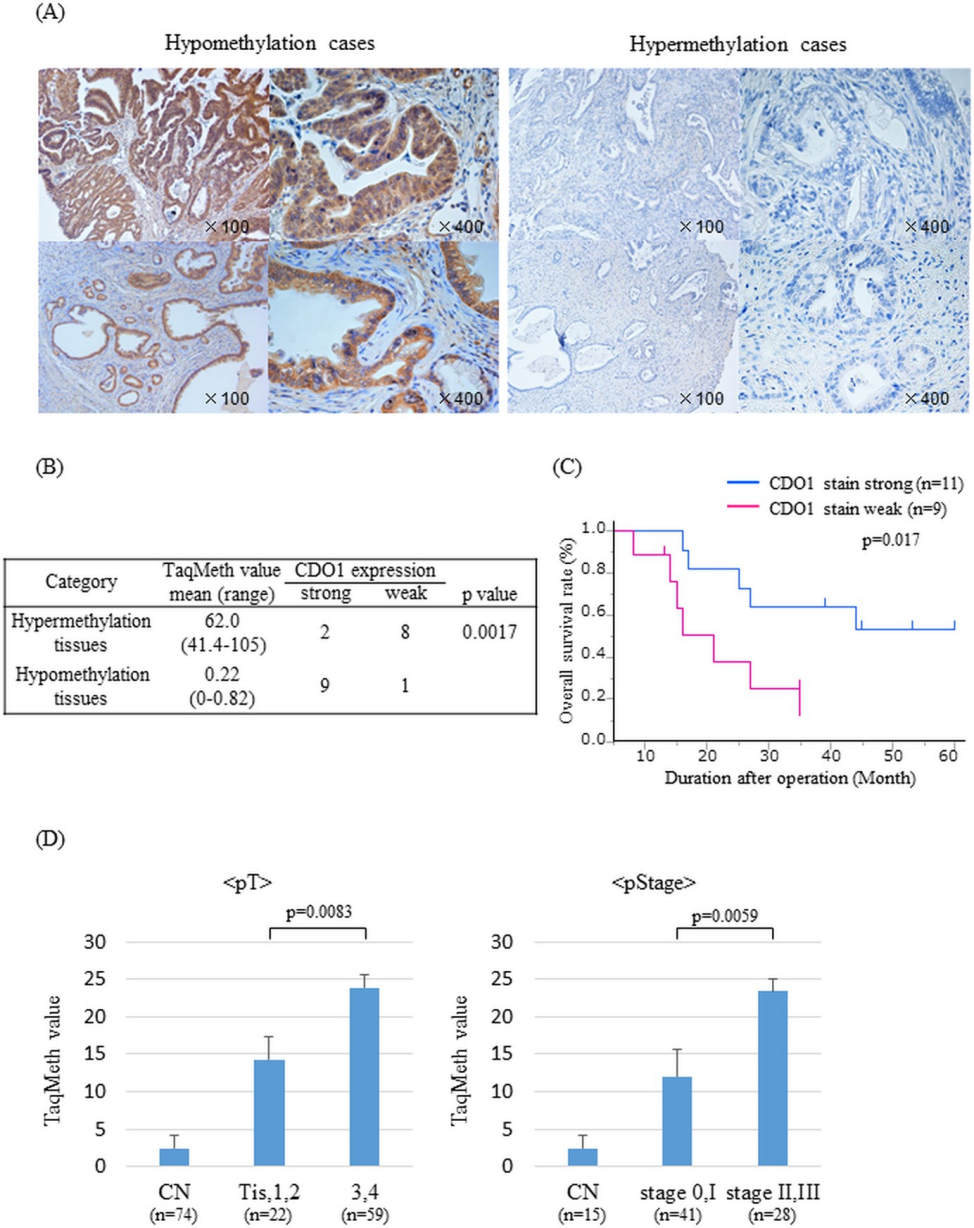
Immunohistochemical staining for CDO1 and correlation of *CDO1* TaqMeth value to clinico-pathological factors in primary tumor. (A) Representative images of immunostaining with an anti-CDO1 antibody in methylation-high or low BTC tumor tissues (original magnification, X100, X400). (B) Correlation between expression of CDO1 (via IHC) and methylation status, (C) and survival. (D) Correlation of *CDO1* TaqMeth value to pT and pStage according to 6th UICC in EHCC. Data are expressed as the mean ±SE.

CDO1 protein expression was examined in the 10 tissues with the highest and lowest of the methylation value using immunostaining with anti-CDO1 polyclonal antibody (Fig 3A). Strong expression of CDO1 protein was observed in 90% of hypomethylation tissues, whereas weak expression was dominant in 80% of hypermethylation tissues. The difference of CDO1 protein expression between these two groups was identified as statistical significance (p = 0.0017) (Fig 3B), suggesting that CDO1 protein expression is significantly associated with promoter DNA methylation of the *CDO1* gene. Additionally, there was also significant difference between CDO1 protein expression and poor survival (p = 0.017) (Fig 3C).

## Discussion

We have discovered that promoter DNA methylation of *CDO1* gene is extra-ordinarily specific to various human cancers by pharmacological unmasking microarray.[6,7] Recently, Andresen and Vedeld et al.[18,19] also reported cancer-specific aberrations of *CDO1* in BTC. However there has been no report on *CDO1* methylation with regard to clinico-pathological relevance in primary BTC, particularly by accurate quantitative assessment. In our current study, we for the first time assessed the clinical and prognostic relevance of *CDO1* methylation status in primary BTC.

Although *CDO1* TaqMeth value was significantly associated with vascular invasion among clinico-pothological factors of total BTC, it tended to increase in a stepwise manner toward higher depth of invasion and higher pathological stage, if restricted to EHCC (Fig 3D). When T factor divided Tis/T1/T2 from T3/T4, and UICCstage divided stage0/I from stageII/III in primary EHCC, there were statistically significant differences between groups, which again recapitulate the results of adenoma-carcinoma sequence of colorectal cancer and those of gallbladder cancer.[12,16]

We have demonstrated that other types of cancer have also exhibited clinical and prognostic relevance of aberrant cancer-specific promoter DNA methylation of *CDO1* gene, such as breast, esophageal, colorectal and gallbladder cancer.[8–10,12,16] Among such well-documented literatures, promoter DNA hypermethylation of *CDO1* reproducibly represents more aggressive phenotypes independently of tumor stage in a multivariate prognostic analysis, and our current study also supported the hypothesis that *CDO1* gene may have predictive value of prognosis in primary BTC.

There is great difference between the cut-off values that differentiated the tumor tissues from the corresponding normal tissues and the most optimal prognostic values. The former one is always lower than the latter one in various cancer such as colon (15.6, 20.5) and gallbladder (5.4, 17.7)[12,16], as in primary BTC (7.2, 28.9). It is hard to believe that a cut-off value discriminating normal and tissue in Taqman assay, which is from Q-PCR analysis, can represent a functional involvement of a protein expression.

Recent studies describing prognostic factors in primary BTC were R0 resection, lymph node metastasis, perineural invasion, portal vein and hepatic artery invasion, and preoperative value of CA19-9 [20–26], and our latest data identified independent prognostic factors of preoperative value of CA19-9 and perineural invasion.[25] However, there has been no established molecular biomarkers to improve management of primary BTC.[27] In this study, *CDO1* methylation was able to clearly stratify the prognostic outcome of EHCC patients after excluding AC using the optimal cut off value of 28.9 according to log-rank plot analysis. Moreover, promoter DNA methylation status of the *CDO1* gene is well associated with CDO1 protein expression in EHCC tumor tissues. These findings indicated that the *CDO1* TaqMeth value of 28.9 has great clinical value to affect prognosis, reflecting its functional contribution in EHCC.

To further clarify the clinical utility in EHCC clinics, we focused on the EHCC patients with low preoperative serum value of CA19-9. Concurrent low group of *CDO1* methylation and CA19-9 exhibited very excellent prognosis, and 5yOS rate reached as much as 89%. On the other hand, both high group and either high group of *CDO1* methylation or CA19-9 level exhibited significantly dismal survival, and the prognosis after surgery remains unsatisfactory with the current treatment of surgery alone. Therefore, these poor groups should consider more effective adjuvant treatment after surgical resection than ever.

Recent study by Andresen et al. has described that cancer detection from ERCP biliary brush samples was robust by using *CDO1* hypermethylation.[17] Furthermore, they rigorously explored epigenetic biomarkers of cholangiocarcinoma, and have identified 13 candidate genes which displayed high methylation frequencies using gene expression profiles of primary cholangiocarcinoma and representative genes methylated across multiple gastrointestinal cancer types.[17–19] The top 5 genes of AUC value were *CNR1P1*, *TMEFF2*, *CDO1*, *MAL* and *SFRP1*, and individual AUC of 0.93, 0.93, 0.91, 0.91 and 0.80, respectively, in the primary tumor tissues, while the best performance was seen in *CDO1* gene in the liquid biopsy (ERCP solution) (AUC = 0.93 as compared to those of other genes, ranged from 0.8 to 0.9). More importantly, *COD1* gene alone is comparable even with the combination analysis (AUC = 0.94) as a single methylation marker. In this study, we didn’t evaluate any other tumor suppressor genes, however we believe that *CDO1* gene would be one of the most excellent cancer-specific biomarkers for diagnostic exploration of BTC at present.

We herein assessed *CDO1* methylation in the tumor tissues and corresponding normal tissues using TaqMeth value as quantitative method.[7] Our quantitative analysis clearly showed high accuracy of diagnosis of cancer tissues from the corresponding normal tissues in AUC of 0.89. In this study, we have to concern that 8 out of 101 corresponding normal tissues were higher methylation than cut off value of 7.2 (this methylation contamination in the normal tissues reduced AUC in our study as compared to Anderson’s data, 0.91). Frequent *CDO1* methylation of the corresponding normal samples from BTC individuals may suggest the existence of a potentially precancerous lesion.

The limitations of this study are a retrospective study design and low statistical power in AC patients. So, this study may suffer from bias, i.e., change in treatment strategy such as surgical procedure, range of lymph node dissection and application of chemoradiotherapy. Prospective validation is thus further needed to clarify the relationship of *CDO1* promoter DNA methylation to prognosis in primary BTC.

In conclusion, we for the first time demonstrated that promotor DNA methylation of *CDO1* gene could be a potential candidate of molecular diagnostic and prognostic biomarker in primary EHCC. *CDO1* methylation status accurately indicates poor prognosis by combination with preoperative serum CA19-9 in the context of the modern treatment strategy. This information would be beneficial to identify patients with recurrence or long-term survival in the outpatient center, and to select the optimal strategies and postoperative surveillance for this type of cancer.

## Supporting information

S1 Table PCR production and sequence of primers and fluorescent probes. (XLSX)

S2 Table Correlation of CDO1 TaqMeth value and clinico-pathological factors in BTC tissues. (XLSX)

S1 Fig The assessment using the log rank plot analysis in ampullary carcinoma. (TIF)
