# Supplementary figures and images for: Methylated promoter DNA of CDO1 gene and preoperative serum CA19-9 are prognostic biomarkers in primary extrahepatic cholangiocarcinoma
Source: PLoS One. 2018 Oct 16;13(10):e0205864. doi: 10.1371/journal.pone.0205864 (PMC6191141; doi:10.1371/journal.pone.0205864)

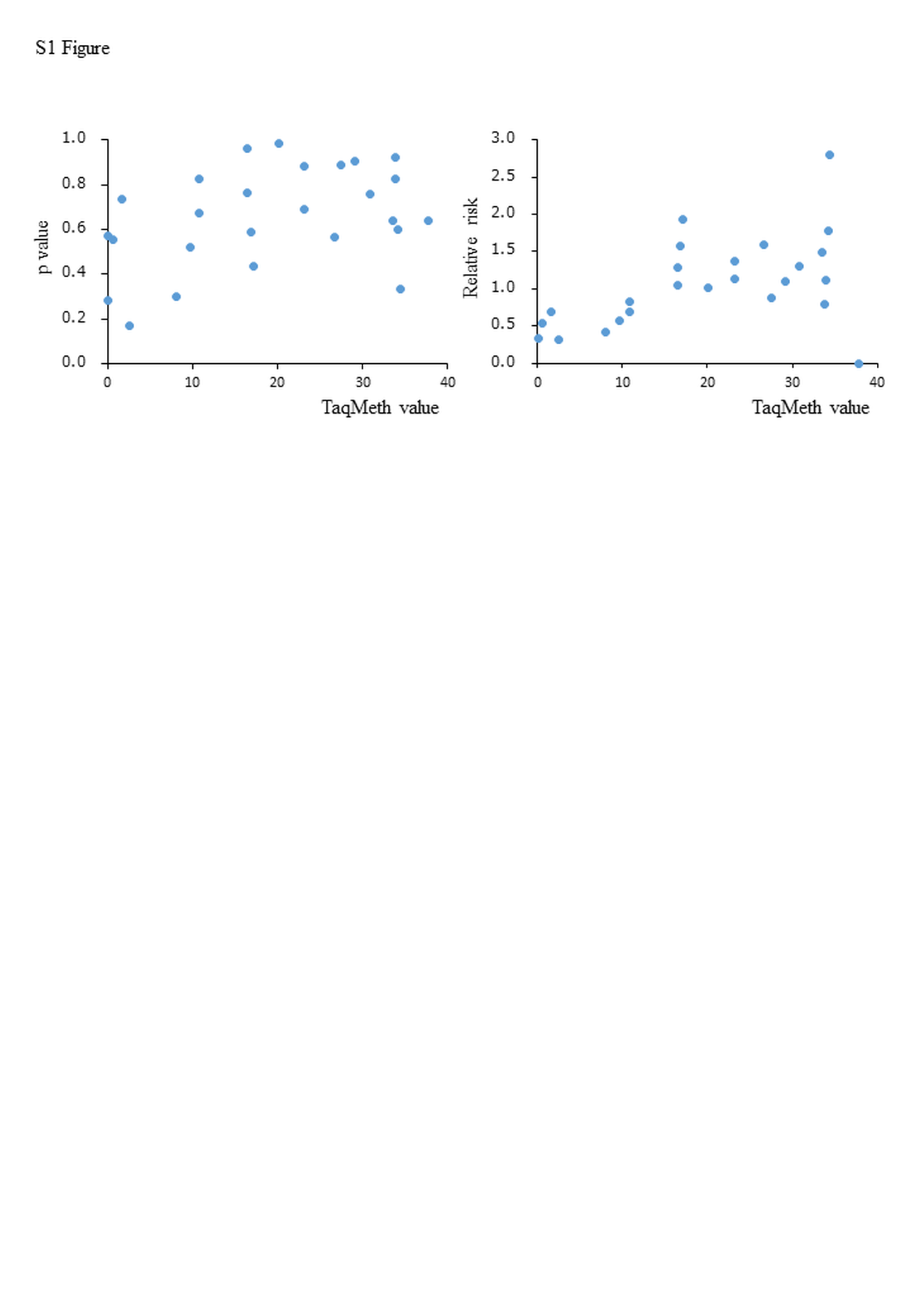

Supplement: S1 Fig — (TIF) [file pone.0205864.s003.tif]
